# Efficacy of a Persian Herbal Remedy and Electroacupuncture on Metabolic Profiles and Anthropometric Parameters in Women with Polycystic Ovary Syndrome: A Randomized Controlled Trial

**DOI:** 10.31661/gmj.v8i0.1389

**Published:** 2019-10-09

**Authors:** Maryam Rouhani, Malihe Motavasselian, Ali Taghipoor, Parvaneh Layegh, Javad Asili, Shokouh Sadat Hamedi, Shapour Badiee Avval

**Affiliations:** ^1^School of Persian and Complementary Medicine, Mashhad University of Medical Science, Mashhad, Iran; ^2^Students Research Committee, Mashhad University of Medical Science, Mashhad, Iran; ^3^Department of Biostatistics and Epidemiology, School of Health, Mashhad University of Medical Sciences Mashhad, Iran; ^4^Department of Radiology, Faculty of Medicine, Mashhad University of Medical Sciences, Mashhad, Iran; ^5^Department of Pharmacognosy, Faculty of Pharmacy, Mashhad University of Medical Sciences, Mashhad, Iran

**Keywords:** Polycystic Ovary Syndrome, Obesity, Insulin Resistance, Phytotherapy, Electroacupuncture

## Abstract

**Background:**

The most prevalent endocrine disorder in women of reproductive age is polycystic ovary syndrome (PCOS). The purpose of this study was to evaluate the efficaciousness of a Persian herbal remedy, as well as electroacupuncture and the combination of them on metabolic profiles and anthropometric parameters in these patients.

**Materials and Methods:**

Eighty overweight women with PCOS were randomly divided into four groups. All of them received metformin 1000 mg and the second group received 5 g of herbal medicine per day (main components: Foeniculum vulgare, Urtica dioica, and Daucus carota), the third group were subjected to 20 electroacupuncture sessions, and the fourth group received both therapies.

**Results:**

After 12 weeks, the body fat and body mass index decreased the most in the herbal medicine+electroacupuncture group, and waist to hip ratio decreased the most in the electroacupuncture group. A significant decrease was also observed in fasting insulin, homeostasis model assessment of insulin resistance. A significant increase was seen in the quantitative insulin sensitivity check index in all intervention groups, but there was no noteworthy difference in these parameters in the control group. Total cholesterol and low-density lipoprotein cholesterol decreased significantly in the electroacupuncture groups and herbal medicine+electroacupuncture. Also, a significant decrease was observed in triglycerides, aspartate aminotransferase, and alanine aminotransferase in the herbal medicine groups and herbal medicine+electroacupuncture.

**Conclusion:**

It is advisable to use this herbal remedy and electroacupuncture for better treatment of metabolic complications and overweight problems in these patients.

## Introduction

The most prevalent endocrine metabolic disorder in women of reproductive age is polycystic ovary syndrome (PCOS) [1-3]. The prevalence of this disease is between 6% and 20% worldwide. In Iran, according to Rotterdam diagnostic criteria (diagnostic criteria for PCOS), the prevalence rate is up to 19.5% [4].

The most significant challenges associated with this disease are metabolic complications and obesity [3, 5]. About 60% of people with this syndrome have a high body mass index (BMI), they are also prone to abdominal obesity, dyslipidemia, hyperglycemia, hypertension (HTN), gestational diabetes mellitus (GDM), type 2 diabetes mellitus (T2DM), and cardiovascular diseases (CVD) [1, 5-7]. It should be noted that these complications lead to increased mortality in women with PCOS in the postmenopausal years [5]. Hyperinsulinemia and insulin resistance (IR) are also chief conditions that are associated with the pathophysiology of PCOS [7], which leads to additional production of androgen and disturbs the process of production and development of ovarian follicles. Consequently, it leads to irregular menstruation and in some cases, infertility [8]. Despite the involvement of several organs and complications, there is still no comprehensive and standard treatment in classical medicine for PCOS [9]. Whereas, the use of complementary therapies such as electroacupuncture and herbal medicine for menstrual and fertility challenges, is increasing, and there are notable studies in this field. For example, one study has recommended traditional Persian treatments for the management of Habs-e-Tams. In traditional Persian medicine (TPM) books, Habs-e-Tams is a condition of prolonged intervals of menstrual bleeding (more than two months). In this study, they have concluded that these treatments are not only effective on irregular menstrual cycles, but they are efficacious on the most obvious symptoms of PCOS, such as associated metabolic dysfunctions of this syndrome. However, it has been suggested that the effectiveness of traditional herbal combinations should be studied in further clinical trials [10]. A review of Persian books and new findings on complementary interventions in this study have shown that the herbal product components based on TPM were effective in reducing BMI, decreasing blood pressure, safeguarding the digestive system and liver, significantly reducing serum leptin, low-density lipoprotein cholesterol (LDL), LDL to high-density lipoprotein (HDL) cholesterol ratio, triglycerides (TG), glucose and serum insulin [11-13]. It was also generally efficacious in increasing menstrual blood flow and folliculogenesis [12-17]. In the current study, the main components of our herbal remedy include Foeniculum vulgare, Urtica dioica, and Daucus carota. A similar herbal combination is used by therapists in TPM for treating PCOS. Studies have shown that triterpenoids and phenolic compounds in F. vulgare stimulate insulin secretion, and have antioxidant properties that catalyze the removal of free radicals and prevent the destruction of liver cells and insulin-secreting cells in the pancreas [18]. Also, this plant is a phytoestrogen that has anti-inflammatory, anti-cancer, and anti-coagulative properties and regulates blood pressure [13]. The essential oil of F. vulgare has a correcting effect on hyperglycemia and serum glutathione activity, as well as on liver enzymes [18]. D. carota is a phytoestrogen that has antioxidant properties and regulates blood pressure as well [19, 20]. Aqueous extract of U. dioica has strong glucose lowering effect, and it decreases body weight, TG, cholesterol, and LDL [12, 21]. It helps to rearrange islets and causes the repairing of pancreatic tissue in diabetic rats [21]. Furthermore, U. dioica extract leads to a decrease in the leptin, insulin, serum glucose, LDL, and LDL/HDL ratio [10]. Also, electroacupuncture has been effective in regulating menstrual periods and decreasing BMI, the waist to hip ratio (WHR), visceral fat and plasma concentrations of leptin, and total cholesterol while increasing insulin sensitivity, LDL, and TG levels [22-25]. Therefore, it is imperative to assess the efficacy of these treatments by performing relevant clinical trials. Hence, in the current study, we have evaluated the efficacy of an herbal remedy obtained from Persian medicine, electroacupuncture, as well as the combination of both therapies as complementary medicine on metabolic profiles and anthropometric parameters in women with PCOS in comparison with the administration of metformin 1000 mg alone.

## Materials and Methods

### Study Design and Sample Size

This study was a randomized clinical trial to evaluate the efficacy of a combination of herbal medicines derived from TPM and electroacupuncture on metabolic and anthropometric indices in patients with PCOS. This study was approved by the Ethics Committee of the Mashhad University of Medical Sciences in the form of a research project (code number: 931324) and was registered in the Iranian Registry of Clinical Trials (registration number: IRCT2015040921671N1). The sample size was computed considering the variance and mean values from similar articles [26] with a 95% confidence and was equal to 15 for each group. However, to increase the accuracy of the study, each group member was increased to 20 [26]. For this purpose, 80 samples were selected from women with PCOS from Persian medicine and Chinese medicine clinics at the Imam Reza Hospital, Mashhad University of Medical Sciences, Iran.

### Diagnostic and Inclusion Criteria

All women who had PCOS between the ages of 15 and 40 years and had a BMI equal to or greater than 25 were enrolled in the study. Diagnostic criteria for this disease were selected according to the 2006 Androgen Excess and Polycystic Ovary Syndrome Society (AE-PCOS), which includes the following:

1. Hyperandrogenism (clinical or paraclinical)

2. Ovarian dysfunction (oligo-anovulation or polycystic ovary in sonographic imagery)

3. Other disorders, which may lead to an increase in androgens or related disorders should be rejected [5, 7, 27]. Clinical hyperandrogenism was considered to be the presence of acne and/or hirsutism and having a score of more than 8 in the modified Ferriman-Gallwey (mF-G) score. Paraclinical hyperandrogenism was also diagnosed based on values ​​higher than normal testosterone and/or androstenedione. Menstrual periods occurring at intervals greater than 42 days or less than six times in a year were considered as oligomenorrhea and the absence of menstrual period or menstrual period occurring less than two times during six consecutive months were considered as amenorrhea. Presence of at least 12 follicles with a diameter of 2 to 9 mm and/or ovarian volume greater than 10 cm3 was considered a positive criterion for diagnosis based on sonographic imagery [5, 7, 27].

### Exclusion Criteria

The exclusion criteria of the study were hyperprolactinemia, the use of hormonal, antidiabetic, antihypertensive, and anticoagulant drugs three months before the start of the study; the presence of a mass in sonographic images, suspected androgen-producing tumor, thyroid dysfunctions, suspected IR syndromes, uncontrolled blood pressure greater than 160/100 mmHg, smoking, hemorrhagic disorders, pregnancy, and lactation.

### Technical Information

The ingredients of the tested herbal medicine were as follows:

1. *F. vulgare:* herbarium number: 38425, common name: *Fennel*, Persian name: *rāzīāna,*

2. *U. dioica:* herbarium number: 45491, common name: *nettle*, Persian name: *Gazaneh,*

3. *D. carota:* herbarium number: 41633, common name: *carrot*, Persian name: *havīj*

4. *Trifolium pratense:* herbarium number: 40926, common name: *red Clover*, Persian name: *Shabdar ghermez*

5. *Curcuma longa:* herbarium number: E1007, common name:* Turmeric*, Persian name:*Zard chobah.*

The mixture was prepared at the Mashhad medicinal herbs market. The herbarium number was obtained from the Faculty of Agriculture of the Mashhad University of Medical Sciences. Microbiology tests were carried out on several samples of the above-mentioned herbal medicine to determine the presence of aerobic mesophilic bacteria, salmonella, E. coli, and other possible pathogens according to the standards of 11804, 9933, and 1810 in the Testa laboratory twice. It was also tested for microbial contamination according to the World Health Organization standard for herbal medicines and was approved. The essential oil obtained was dried over anhydrous sodium sulfate, and then analyzed employing the as gas chromatography (GC) and GC- mass spectrometry (GC-MS) technique. The GC analysis was performed using a Varian CP-3800 GC equipped with an FID detector, fused-silica column (CP-Sil 8CB, 50 m × 0.25 mm, film thickness, 0.12 m). The GC-MS analyses were performed using an Agilent 5975 apparatus with an HP-5ms column (30 m x 0.25 mm i.d., 0.25 µm film thickness) interfaced with a quadruple mass detector. Also, trans-anethol was identified as the most important compound present in the extracted essence after undergoing the standardization procedure at the Faculty of Pharmacy, Mashhad University of Medical Sciences, Iran. The drug was registered by the Patent and Intellectual Property Organization in Iran (approval number: 139650140003004422) and was scientifically approved by the Research and Technology Unit of the Mashhad University of Medical Sciences. For electroacupuncture, Huan-Qiu (single use steel needles, length: 30-50 mm, diameter: 0.30mm) were employed, and in all groups, 500 mg metformin hydrochloride enteric-coated tablets manufactured by Aria Pharmaceutical Company (approval number: 9609356) were used.

### Data Collection

Height and weight of the patients were measured without shoes in the morning. Weight, BMI, and anthropometric measurements of the body were recorded by the Gaia 359 (Body Composition Analyzer System) at the beginning and end of the treatment. Hip circumference was measured at the level of the major trochanters through the pubic symphysis, and waist circumference was measured midway between the lateral lower rib margin and the iliac crest to the nearest ± 0.1 cm employing a scale. To prevent errors, all measurements were done by the same person. Venous blood samples were taken in the second day of the menstrual cycle, twice after a 12-hour fasting period (from 08:00 AM to 09:00 AM) during the study (at the beginning and 12 weeks later). The metabolic parameters assessed included fasting plasma glucose, aspartate aminotransferase (AST), alanine aminotransferase (ALT, Pars Azmoon Co., Tehran, Iran), total cholesterol, TG (Man Lab, Tehran, Iran), LDL-C, and HDL-C (PISHTAZTEB, Tehran, Iran). They were measured enzymatically using commercial kits in the special medical laboratories of Imam Reza Hospital (Mashhad University of Medical Sciences, Mashhad, Iran). Fasting serum insulin was measured by the Insulin IRMA kit purchased from IZOTOP company (H-1121 Budapest, Hungary). We also computed the homeostatic model assessment of IR (HOMA-IR), homeostatic model assessment of beta cell function (HOMA-β), and the quantitative insulin sensitivity check index (QUICKI) to assess insulin sensitivity and insulin secretion [28-30]. HOMA-IR was computed as fasting insulin (μU/mL) × fasting glucose (mg/dl) / 405. HOMA-β was calculated as fasting insulin (μU/ml) × 360 / fasting glucose (mg/dl) − 63. QUICKI was calculated as 1 / (log fasting insulin [μU/mL] + log fasting glucose [mg/dl]). Then in the clinical stage, the patient’s history (i.e., demographic information and signs and symptoms associated with the disease) was obtained and recorded. At the end of the treatment, an overview of the process was done to assess the complications associated with the patient’s treatment.

### Interventions

The patients were randomly assigned into four groups (20 individuals each). Before randomization, the participants were matched according to their menstruation time and their BMI. Matching was done using menstrual dysfunction (amenorrhea, oligomenorrhea) and BMI. The random numbers table was used for random allocation. The research assistant, who was blind to the interventions and allotted numbers, assigned the participants to the study interventions. Then, an informed consent form was completed by the patients after explaining their treatment methods. For individuals under 18 years old, written consent was received from their parents and attached to the consent letter. The TPM approach to the treatment of multiple organ diseases emphasizes attention to be paid to an individual’s dietary requirements [31]. For this reason, in this study, along with the interventions, the same dietary recommendations were employed in all groups [32-34]. Each of the four groups received two pills of metformin 500 mg daily. The second group was given granulated herbal medicine in a sachet (5 g) daily, the third group was subjected to 20 sessions of electroacupuncture (two sessions in a week at the first and second month and one session in the third month), and the fourth group received a combination of both treatments in a 12-week period. Acupuncture points (identified by Liangmen [ST-21], Tianshu [ST-25], Shuidao [ST-28], Guilai [ST-29], Zhangmen [liver13], and Abd Zigong) on both sides of the body were selected. Acupuncture points [identified by Zhongwan [REN-12], Qihai [REN-6], Guanyuan [REN-4], Yinlingquan [SP-9], Sanyinjiao [SP-6], and Fenglong [ST-40]) on both lower legs were also selected [35]. All needles were inserted to a depth of 15 to 35 mm after sterilization and were rotated by hand after inserting to induce the de qi sensation (a sense of heaviness or numbness in the area surrounding the site of insertion). To stimulate the ST28, ST25, and liver 13 points, the electrodes (KWD-808 pulse acupuncture and moxibustion treatment device) were connected to the needles and employed at a frequency of 2Hz. The degree of stimulation intensity was such that it induced muscle contractions at the site without any pain or discomfort. The needles that were not attached to the electrode were rotated manually every 10 minutes to induce the de qi sensation. The needles were left for 30 minutes. Besides, patients were educated to slow down on their eating habits as much as possible and eliminate foods with low nutritional value, as well as avoid excessive consumption of food prior to participation in the study. Recommended foods for all patients were similar in terms of nutritional requirement. The types of food were extracted from the Persian medical reference books according to dietary recommendations. Patients were visited once a week to ascertain compliance with dietary recommendations, as well as examine the possible side effects of the treatment. Also, during the three months of treatment, the patients did not use any other medication or supplement in addition to the treatment they received.

### Statistical Methods

Data obtained from clinical and paraclinical observations were analyzed by SPSS software (version 16, Chicago, Illinois, USA). Initially, the normal distribution of the quantitative variables was evaluated using the one sample Kolmogorov-Smirnov test. The chi-square test was employed to compare the qualitative variables in the four groups, respectively. For comparison of quantitative variables before and after the study within each group, the paired samples t-test was employed. In the absence of normal distribution, the Wilcoxon non-parametric test was applied, and to compare the variations among the four groups, the one-way ANOVA or Kruskal–Wallis test was used. P-value<0.05 was considered statistically signiﬁcant in all analytical tests.

## Results

**Figure 1:**
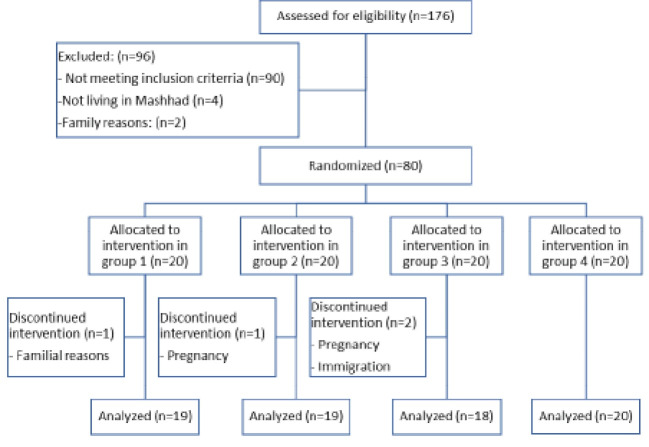
CONSORT flow diagram of study

At the start of this study, 80 people were included according to the inclusion criteria, out of which one due to family reasons, two due to pregnancy, and one due to immigration did not complete the trial. Hence, 19 people in group 1 (control), 19 in group 2 (herbal medicine), 18 in group 3 (electroacupuncture), and 20 in group 4 (herbal medicine + electroacupuncture) completed the trial (Figure-1). More than 90% of the patients took metformin tablets regularly, and about 30% reported digestive problems such as bloating or nausea due to its use. Herbal medicine and (herbal medicine + electroacupuncture) groups did not report any specific adverse effects associated with the use of the herbal medication packaged in a sachet. The mean age of 76 female participants was 27.65 ± 6.7 years, out of which, 21 (26.9%) were single, and 57 (73.1%) were married. The age distribution of the patients in the four groups was normal (P=0.065). All anthropometric and laboratory parameters other than HDL, AST, and ALT produced a normal distribution curve in all four groups. Comparison of the four groups showed that anthropometric parameter and mean age were not signiﬁcantly different before the intervention (Table-1 and 2). In this trial, after 12 weeks of intervention, the anthropometric variables including BMI, body fat (BF), and WHR in each of the four groups reduced significantly when compared with the initial values. However, the highest reduction in BMI and BF levels were observed in the (herbal medicine + electroacupuncture) group, and the highest difference in WHR was observed in the electroacupuncture group (Table-2). Also, fasting insulin and HOMA-IR decreased significantly in all the intervention groups. The highest insulin reduction was observed in the electroacupuncture group, and the highest reduction in HOMA-IR was seen in the herbal medicine group and electroacupuncture group, respectively. However, in the control group, there was no significant difference in these parameters. There was also a significant decrease in HOMA-β in the herbal medicine group and electroacupuncture group, while in the control and (herbal medicine + electroacupuncture) groups, no significant change was observed. Significant increase in QUICKI was observed in all intervention three groups, with the highest increase observed in the electroacupuncture group, but in the control group, no significant change was observed (Table-2). Total cholesterol and LDL in both the electroacupuncture and (herbal medicine+electroacupuncture) groups showed a significant decrease at the end of treatment compared to the beginning of treatment. The highest difference in total cholesterol was observed in the (herbal medicine + electroacupuncture) group, and the highest reduction in LDL was observed in the electroacupuncture group. Although differences in group 4 were noticeable, no significant decrease was observed in the control group and the herbal medicine group. A significant decrease in TG, AST, and ALT were observed in the herbal medicine and (herbal medicine + electroacupuncture) groups, and the highest difference was observed in the (herbal medicine + electroacupuncture) group, while no significant changes were observed in the control and electroacupuncture groups. HDL was lower in all the groups at the end of treatment, although this change was not significant in any of the groups. The LDL to HDL ratio increased slightly in the control and herbal medicine groups at the end of treatment, but the increase was not significant. In the electroacupuncture and (herbal medicine + electroacupuncture) groups, this ratio decreased significantly (Table-2). In comparison with the control group, according to the within-group changes, a significant reduction was observed in BMI in the (herbal medicine + electroacupuncture) group (P=0.012); HOMA-IR in the electroacupuncture group (P=0.045); AST in the herbal medicine group (P=0.034) and (herbal medicine + electroacupuncture) group (P=0.006); and ALT in the electroacupuncture group (P=0.05) and (herbal medicine + electroacupuncture) group (P=0.01). Also, a significant reduction was seen in WHR in the electroacupuncture group (P=0.049), but an insignificant reduction was observed in the (herbal medicine + electroacupuncture) group (P=0.059) and in the electroacupuncture group (P=0.06), which were clinically important. A significant difference was not reported in the other anthropometric and laboratory variables (Table-2).

## Discussion

This study was conducted to evaluate the efficaciousness of a Persian herbal remedy and electroacupuncture as complementary therapies on metabolic profiles and anthropometric parameters in women with PCOS.

### Anthropometric Parameters

In relation to the above, the combination of herbal medicine and electroacupuncture has a significant and distinct statistical and clinical difference compared to the other groups in reducing BMI and BF. Therefore, it can be used to achieve a better result for these parameters in these patients. Overweight and obesity, which increase complications in PCOS patients, are seen in about 60% of people [1, 5, 7]. Hence, it is imperative to have more effective treatment options for overweight patients.

In this study, the results indicate the beneficial effect of electroacupuncture in the reduction of abdominal fat. Abdominal obesity is associated with an increased risk of cardiovascular diseases and diabetes [1, 7]. WC is related to the amount of subcutaneous fat and, as mentioned in numerous studies, the main functions of body electroacupuncture are lipolytic activity and its effect in increasing the metabolism of lipids [25, 36]. Also, the mechanism of the effect of phytoestrogens (like our herbal remedy) on metabolic syndrome was addressed in five main dimensions, in which two of them were increasing basic metabolism, energy consumption, and burned fat [37].

### Metabolic and Lipid Profiles

One of the most important findings of this study is the role of the above treatments in IR. In other words, the effect of this herbal remedy and electroacupuncture was highly significant in reducing IR and increasing its secretion in overweight patients with PCOS. IR is one of the main pathophysiological causes of PCOS, which itself leads to being overweight and many other complications associated with the disease, such as the interruption of ovulation and increased male hormones and clinical manifestations [38-41]. Considering the above results, the use of the studied herbal remedy or electroacupuncture has had a positive impact on one of the most important parameters associated with PCOS although the combination of these two treatments does not increase the efficacy of the treatment on insulin-associated parameters. As indicated in the results, the highest changes in cholesterol were observed in group 4 (herbal medicine + electroacupuncture), which shows a positive effect of the combination of herbal medicine and electroacupuncture in reducing cholesterol. Also, reduction in TG, AST, and ALT were significant in the herbal medicine group and highly significant in the (herbal medicine + electroacupuncture) group, but there was no significant change in these factors in the control and electroacupuncture groups. Regarding the prevalence of metabolic syndrome, hyperlipidemia, fatty liver, and liver disorders in PCOS patients [1], prevention and control of hyperlipidemia, as well as the adoption of preventive measures against cardiovascular diseases, are among the most important guidelines in managing patients with PCOS. The above changes also indicate the positive effect of our herbal remedy in reducing TG and improving liver function, but electroacupuncture can also increase this effect with positive interaction with herbal medicine. In a clinical trial by Hu *et al*. (2014), administration of 1500 mg of metformin for twelve weeks to 40 patients with PCOS resulted in a significant decrease in the index of IR and BMI in overweight people [42]. Meanwhile, in our study, metformin 1000 mg, which was administered to group 1 (control) per day, did not have a significant effect on the reduction of markers of IR and with an increase in the dose, more gastrointestinal complications were observed in our patients. In a study conducted by Mhaidat *et al*. on rats model, it was noted that F. vulgare was effective in preventing an increase in blood glucose, and reduced high cholesterol, ALT, AST, urea, and creatinine in diabetic rats [18]. In a review study published by Jungbauer *et al*. in 2014, the mechanism of the effect of phytoestrogens on metabolic syndrome was addressed in five main dimensions, of which the most important were increased insulin sensitivity. In this study, the extract of red clover, licorice, and soybeans was identified as an effective ingredient in the treatment of PCOS [37]. In another study by Kianbakht *et al*., on the effect of U. dioica leaf extract on 46 patients with T2DM, it was observed that there was a significant reduction in fasting glucose levels, but no significant effect was reported on AST and ALT levels [12]. In another clinical trial study performed by Chedraui *et al*. on 60 postmenopausal women over 40 years with oral administration of 80 mg of red clover for three months, it was found that this extract significantly reduced the levels of TG, LDL, and lipoprotein A in women with a BMI greater than 25 [43]. In another study by Soni *et al*. done on ten healthy volunteers, who were given 500 mg of curcumin daily for seven days, it was found there was a 33% decrease in LDL oxidation, 11% reduction in total cholesterol, and 29% increase in the concentration of HDL [44]. However, in contrast to the study of Kianbakht *et al*., with this herbal medicine, a significant reduction in the ALT and AST levels, as well as an increase in sensitivity to insulin, were observed, which may be due to the effects of other components or their synergistic effects. However, it is worth noting that although the drug resulted in a decrease in LDL and total cholesterol, as reported in the above studies, this decrease was not statistically significant in the herbal medicine group. However, in this study, there was also a significant decrease in TG, which was directly linked to the use of herbal medicine. In another review study by Stener-Victorin *et al*. on the effects of electroacupuncture on PCOS patients, this treatment was shown to stimulate weight loss and increase insulin sensitivity [22]. In a study by Zheng *et al*. in 2013 on 86 PCOS patients, it was observed that electroacupuncture had more of an effect on BMI and WHR compared to the administration of 1500 mg metformin daily, and it had fewer side effects [23]. In a study by Tugrul *et al*. on 55 obese and overweight women, it was found that electroacupuncture in some parts of the body and ear for 20 consecutive days had a significant effect on weight loss, total cholesterol, LDL, and TG in patients with PCOS [24]. In another study, conducted by Abdi *et al*. on 196 overweight individuals, after six weeks of body electroacupuncture, there was a significant reduction in the anthropometric and lipid profile in both the authentic and sham groups [36]. Our study also confirmed the significant effect of electroacupuncture on the reduction of anthropometric and lipid profile. It is important to note that in this study, there was a significant decrease in the ratio of LDL to HDL in the electroacupuncture groups and (herbal medicine + electroacupuncture) groups. Based on these findings, electroacupuncture is recommended in patients with hyperlipidemia or who are susceptible to hyperlipidemia. One of the most valuable points in this study is the investigation of the effect of the above herbal medicine and the efficacy of combining this drug with electroacupuncture on anthropometric and metabolic variables in patients with PCOS for the first time. Moreover, none of the patients reported a specific complication regarding the use of this herbal medicine or electroacupuncture during treatment. There were also some limitations in this study, which could be considered in subsequent studies. For example, we did not use any placebo or sham acupuncture because the Ethics Committee did not allow us to do so. Also, the duration of the intervention was limited to 12 weeks. Therefore, further follow-up and at least a six-month period of treatment may lead to further changes. Also, it seems that other factors such as HbA1C and very-low-density lipoprotein should be evaluated; however, they were not investigated in this study because of cost limitations. Finally, the abnormal distribution of HDL, AST, and ALT, were limitations as well.

## Conclusion

The combination of the proposed herbal remedy, body electroacupuncture, and metformin has been significantly effective in reducing weight, abdominal fat, TG, and liver enzymes. Moreover, the herbal remedy, as well as body electroacupuncture, is useful for reducing IR and increasing its secretion. Significant changes were observed in lipid profiles with electroacupuncture as well as its incorporation into an herbal remedy. Consequently, it seems that considering the results of the current study and its consonance with the findings of previous studies, it is advisable to use complementary medicine including the above herbal remedy or electroacupuncture in the treatment of PCOS patients. Although the combination of these treatments is more effective, further studies are needed to obtain more accurate results.

## Acknowledgment

This article was part of the results of a thesis for the doctoral degree of Maryam Rouhani, in Persian Medicine at the Mashhad University of Medical Sciences. We wish to thank the Vice-Chancellor for Research of the Mashhad University of Medical Sciences to accompany and grant assignment (grant number: 931324) and also all the professors and scholars who were involved in this project.

## Conflict of Interest

None.

**Table 1:** General Characteristics of Participants at Baseline Study. Data Presented as Means ± SD

**Variables**	**Group 1** **(n=19)**	**Group 2** **(n=19)**	**Group 3** **(n=18)**	**Group 4** **(n=20)**	**P-value** ^*^
**Age(year)**	27.42 ± 6.09	26.36 ± 6.99	29.82 ± 8.23	27.45 ± 5.46	0.45
**BMI** ^*^ **(kg/m** ^2^ **)**	31.23 ± 4.50	31.01 ± 4.81	32.07 ± 4.86	31.71 ± 5.09	0.90
**BF (kg)**	30.75 ± 6.87	29.91 ±7.47	32.67 ± 8.72	30.41 ± 8.48	0.42
**WHR (cm)**	0.97 ± 0.06	0.99 ± 0.06	1.02 ± 0.06	1.00 ± 0.05	0.17

**BMI:** Body mass index; **BF:** Body fat; **WHR:** Weight to hip rate; ^*^Obtained from One-Way ANOVA test

**Table 2:** Comparison of Clinical and Biochemical Characteristics of Participants

	**Group1 ( n=19 )**			**Group4 ( n=19 )**			**Group3 ( n=18 )**			**Group4 ( n=20 )**		
	**Baseline**	**Weeks 12**	**P** ^*^	**Baseline**	**Weeks 12**	**P** ^*^	**P-value** ^**^	**Baseline**	**Weeks 12**	**P** ^*^	P-value**	**Baseline**	**Weeks 12**	** P** ^*^	P-value**
**BMI** **(kg/m** ^2^ **)**	31.23±4.50	30.05±4.79	0.001	31.01±4.81	29.65± 5.19	0.001	0.660	32.07±4.86	30.47± 4.57	0.001	0.335	31.71± 5.09	29.44± 4.89	0.001	0.012
**BF** **(kg)**	30.75± 6.87	28.38±7.09	0.001	29.91±7.47	27.68± 7.77	0.001	0.862	332.67±8.72	29.73± 7.96	0.001	0.499	30.41± 8.48	26.76± 8.48	0.001	0.115
**WHR (cm)**	0.98 ± 0.06	0.96 ± 0.05	0.036	0.99 ± 0.06	0.97 ± 0.05	0.001	0.285	1.02± 0.07	0.99 ± 0.05	0.001	0.049	1.00± 0.05	0.98 ± 0.05	0.001	0.059
**FBS (mg/dl)**	95.10± 8.19	93.26± 9.06	0.298	98.27±10.75	93.38±22.34	0.281	0.474	95.04±5.41	90.11± 7.59	0.034	0.496	98.60±11.13	95.05± 9.58	0.162	0.694
**Insulin (pmol/l)**	10.93± 5.91	9.41 ± 5.96	0.945	10.96± 4.45	7.91 ± 3.47	0.003	0.340	11.35± .86	6.57 ± 3.71	0.001	0.060	10.02± 6.23	7.14 ± 3.46	0.008	0.406
**HOMA-IR**	2.54 ± 1.34	2.19 ± 1.46	0.147	2.54 ± 1.34	2.19 ± 1.46	0.147	0.234	2.65± 1.35	1.45 ± 0.85	0.003	0.045	2.51± 1.73	1.72 ± 0.95	0.008	0.265
**HOMA-β**	134.23±87.19	117.81±76.28	0.107	126.58±91.66	81.71±59.57	0.006	0.190	138.81±80.06	96.43±64.52	0.019	0.364	101.87±56.61	80.51±33.40	0.167	0.823
**QUICKI**	0.34 ± 0.02	0.37 ± 0.09	0.159	0.33 ± 0.02	0.37 ± 0.06	0.004	0.774	0.34 ± 0.04	0.41 ± 0.16	0.001	0.160	0.35± 0.04	0.36 ± 0.04	0.009	0.677
**Total cholesterol (mg/dl)**	167.31±26.21	160.63±25.53	0.266	181.81±26.53	177.09±25.84	0.225	0.787	190.41±33.72	176.41±36.36	0.018	0.347	202.15±37.74	183.00±35.53	0.005	0.097
**TG (mg/dl)**	119.26±48.44	102.94±38.54	0.133	128.40±63.52	103.95±40.44	0.035	0.591	117.11±53.03	123.47±60.89	0.514	0.162	150.50±85.11	113.80±57.49	0.007	0.190
**LDL-C (mg/dl)**	109.57±20.95	103.94±25.65	0.292	120.95±23.76	119.27±23.94	0.647	0.173	127.13±30.60	112.88±34.64	0.013	0.956	134.35±32.13	122.80±33.74	0.026	0.152
**HDL-C (mg/dl)**	42.57±11.23	39.21± 7.88	0.629	43.05± 8.35	41.95±9.75	0.404	0.502	50.87±17.63	45.35±7.38	0.083	0.551	45.45±9.08	44.50± 6.92	0.353	0.485
**LDL-C / HDL-C**	2.69 ± 0.70	2.75 ± 0.89	0.728	2.89 ± 0.72	2.95 ± 0.82	0.663	0.983	2.77±1.17	2.56±0.97	0.025	0.181	3.06 ± 0.98	2.83± 0.98	0.030	0.134
**AST (U/L)**	18.68± 4.61	21.73±18.07	0.716	20.31± 7.08	17.13±4.52	0.015	0.034	23.15±11.82	16.64±3.84	0.092	0.071	20.31± 7.08	17.85±9.29	0.001	0.006
**ALT (U/L)**	22.63±10.24	25.31±25.28	0.864	21.09± 8.87	25.00±11.14	0.008	0.069	23.47±10.90	18.58±5.93	0.068	0.050	23.10±10.22	16.15±7.49	0.001	0.010

test vs. baseline, ^**^ Obtained from One-Way ANOVA testvs. group1;** BMI:** Body mass index;**BF:** Body fat; **WHR:** Weight to hip rate; **FBS:** Fasting blood sugar; **HOMA-IR:** Homeostasis model of assessment-insulin resistance; **HOMA-β:** Homeostatic model assessment-beta-cell function; **QUICKI:** Quantitative insulin sensitivity check index; **TG:** Triglycerides; **LDL-C:** Low-density lipoprotein cholesterol; **HDL-C:** High-density lipoprotein cholesterol; **AST:** Aspartate aminotransferase; **ALT:** Alanine aminotransferase.
